# DNA Methylation and Hydroxymethylation in Primary Colon Cancer and Synchronous Hepatic Metastasis

**DOI:** 10.3389/fgene.2017.00229

**Published:** 2018-01-09

**Authors:** Silvia Udali, Domenica De Santis, Andrea Ruzzenente, Sara Moruzzi, Filippo Mazzi, Greta Beschin, Stephanie A. Tammen, Tommaso Campagnaro, Patrizia Pattini, Oliviero Olivieri, Alfredo Guglielmi, Sang-Woon Choi, Simonetta Friso

**Affiliations:** ^1^Department of Medicine, School of Medicine, University of Verona, Verona, Italy; ^2^Department of Surgery, School of Medicine, University of Verona, Verona, Italy; ^3^Friedman School of Nutrition Science and Policy, Tufts University, Boston, MA, United States; ^4^Chaum Life Center, CHA University, Seoul, South Korea

**Keywords:** epigenetics, methylcytosine, hydroxymethylcytosine, primary colon cancer, synchronous hepatic metastasis

## Abstract

Colon cancer is one of the most frequent solid tumor and simultaneous diagnosis of primary colon cancer and liver metastases occurs in about one fourth of cases. The current knowledge on epigenetic signatures, especially those related to hydroxymethylation in primary cancer tissue, synchronous metastasis, and blood circulating cells is lacking. This study aimed to investigate both methylcytosine (mCyt) and hydroxymethylcytosine (hmCyt) status in the DNA of individual patients from colon cancer tissue, synchronous liver metastases, and in cancer-free colon and liver tissues and leukocytes. Patients undergoing curative surgery (*n* = 16) were enrolled and their laboratory and clinical history data collected. The contents of mCyt and hmCyt were determined by a liquid chromatography/mass spectrometry (LC/MS/MS) method in DNA extracted from primary colon cancer, synchronous hepatic metastatic tissues and homologous cancer-free tissues, i.e., colon and liver tissues as well as leukocytes. The mCyt and hmCyt levels were compared between cancerous and cancer-free tissues, and correlations between leukocytes and colon/liver tissues for both the mCyt and hmCyt levels were evaluated. The mCyt levels were similar in primary colon cancer and liver metastasis tissues (4.69 ± 0.37% vs. 4.77 ± 0.38%, respectively, *p* = 0.535), and both primary and metastatic tissues were hypomethylated compared to cancer-free colon (4.98 ± 0.26%). The difference in the mCyt content between cancerous and cancer-free colon tissues was significantly lower in primary colon cancer (*p* = 0.004), but not in liver metastasis (*p* = 0.148). The hmCyt content was similar in primary colon cancer compared to liver metastasis (0.035%, C.I. 0.024–0.052% versus 0.035%, C.I. 0.021–0.058%, respectively, *p =* 0.905) and markedly depleted compared to the cancer-free colon (0.081%, C.I. 0.055–0.119%) with a statistically significant difference (*p* < 0.05) for both comparisons. The mCyt levels showed a borderline correlation between leukocytes and colon cancer tissue (Pearson’s correlation coefficient = 0.51, *p* = 0.052) while no correlations were detected for the hmCyt levels. In conclusion, primary colon cancer and synchronous liver metastasis tissues showed a similar epigenetic status but were significantly hypomethylated and hypohydroxymethylated as compared to homologous cancer-free colon tissues.

## Introduction

Colorectal cancer is the third most frequently diagnosed cancer in males and the second in females (Jemal et al., 2011) and liver tissue is the most common site of colorectal cancer metastasis, observed in up to one fourth of patients at the time of initial diagnosis (Silberhumer et al., 2016).

Various studies on the pathogenesis of colorectal cancer and cancer development suggest that, in addition to genetic modifications, there is an important role for epigenetic markers that occur early and manifest frequently in this type of cancer (Okugawa et al., 2015). Epigenetics refers to the complex of heritable and potentially reversible mechanisms that regulate gene expression without alterations in the DNA sequence, including DNA methylation, histone modifications and microRNAs (Wolffe and Matzke, 1999; Bird, 2007; Carthew and Sontheimer, 2009), all of which are frequently studied phenomena in cancer (Feinberg and Tycko, 2004; Ehrlich, 2006).

One of the most extensively evaluated epigenetic phenomenon in cancer is DNA methylation, consisting of the transfer of a methyl group (-CH3) to the 5′ position of a cytosine at CpG dinucleotide residues. This process can modulate gene expression by modifying the accessibility of DNA to transcriptional machinery (Jones and Takai, 2001; Heyn and Esteller, 2012). Epigenetic modifications in colon cancer consist of gene-specific DNA hypermethylation and DNA hypomethylation, i.e., global hypomethylation, as well as long interspersed nucleotide element-1 (LINE-1) and oncogene hypomethylation (Vaiopoulos et al., 2014). Currently, significant attention has been devoted to the gene-specific hypermethylation in colon cancer that is responsible for the silencing of specific genes, such as tumor-suppressor genes (Baylin and Jones, 2011; Ng and Yu, 2015). Nevertheless, the first evidence of aberrant DNA methylation in colon cancer dates back to Goelz et al. (1985) with a report on global DNA hypomethylation in tumor tissues. Global DNA hypomethylation is thought to play an important role in carcinogenesis by inducing chromosomal instability and the global loss of imprinting (Gaudet et al., 2003; Holm et al., 2005; Wilson et al., 2007; Ehrlich and Lacey, 2013).

Recently, 5-hydroxymethylcytosine (hmCyt) has been recognized as a new epigenetic marker that is generated by the oxidation of methylcytosine (mCyt) catalyzed by methylcytosine oxygenase TET1 (Kriaucionis and Heintz, 2009; Tahiliani et al., 2009; Munzel et al., 2011; Shukla et al., 2015). Hydroxymethylcytosine has been proposed as an intermediate product of the DNA demethylation process (Globisch et al., 2010; Wu and Zhang, 2014), but growing evidence suggests that hmCyt could be more than an intermediate of DNA demethylation and could have an additional role in transcriptional regulation by attracting or repelling specific DNA-binding proteins (Ye and Li, 2014; Liu et al., 2016). Moreover, a specific role in the regulation of gene expression during colonocyte differentiation and in human colon cancer has been recently described (Chapman et al., 2015).

We previously observed that a reduction of hmCyt content is a peculiar characteristic of primary liver cancer tissues compared to non-neoplastic liver tissue. DNA hypomethylation is present in hepatocellular carcinoma but not in cholangiocarcinoma tissues (Udali et al., 2015). These data suggest that the epigenetic landscape, as it refers to global methylation and hydroxymethylation status, could depend on cancer type and on whether the tissue pertains to primary neoplastic or metastatic lesions. The aim of the present study was to quantify mCyt and hmCyt levels by an LC–MS/MS method in patients affected by metastatic colon cancer, by evaluating primary colon cancer tissue, synchronous hepatic metastasis tissues and in homologous cancer-free tissues, i.e., colon, liver and leukocyte DNA, the latter of which to determine whether the status of these epigenetic markers in leukocyte DNA could be a putative biomarker for cancer in specific tissues.

## Materials and Methods

### Study Subjects

The study subjects were 16 patients, 9 males and 7 females, of a mean age of 66 years who underwent curative surgery intervention for colon cancer and synchronous liver metastasis resection. Patients were enrolled from November 2009 to May 2012 at the Division of General and Hepatobiliary Surgery of the Verona University Hospital (Verona, Italy). Surgical resectability criteria were preserved liver function, a class A Child-Pugh score and the absence of extrahepatic metastases. Exclusion criteria were the presence of relevant concurrent medical conditions, such as acute or chronic diseases. Patients undergoing B vitamin supplementation and/or those on a pharmacological therapy that could interfere with DNA methylation pathways were excluded. The selection of patients in such clinical conditions to undergo a surgical intervention, excluded the analysis of subjects with a more widespread metastatic disease. The study was approved by the Institutional Review Board Ethical Committee of the University of Verona School of Medicine Hospital (Study number ET 001). Written informed consent was obtained from each patient after a detailed explanation of the study, in accordance with the Declaration of Helsinki.

### Biochemical Analysis and Clinical Data Collection

Each patient underwent blood chemistry tests including a complete blood count, C-reactive protein (CRP), glucose, aspartate transaminase (AST), alanine transaminase (ALT), alkaline phosphatase (ALP), cholinesterase (CHE), gamma-glutamyl transpeptidase (GGT), total and direct bilirubin, and international normalized ratio (INR). Serum levels of B vitamins (folate, vitamin B6, and vitamin B12), homocysteine and ferritin were also assessed. Furthermore, a detailed clinical history data and lifestyle habits were recorded by a trained physician. As for smoking, patients were distinguished between former and current users.

### Blood and Tissue Sample Collections and DNA Extraction

From each patient, blood and tissue specimens were collected for epigenetic analyses. Tissue samples of primary colon cancer (CCr) and synchronous liver metastasis (LM) were collected together with homologous cancer-free colon (C) and cancer-free liver (L) specimens. The cancer-free tissues were obtained from a surgical biopsy of a region far from the cancer tumor mass. A histological diagnosis of all the collected tissues was then performed by a pathologist blinded to patient participation to the study, and cancer-free tissues, i.e., colon and liver, were confirmed to be negative for the presence of neoplastic cells. The histological analysis identified colon cancer specimens as adenocarcinomas among which 14 were low grade and 2 high grade. The tissue samples were collected immediately after surgical excision, sliced into aliquots of approximately 100 mg, snap-frozen in liquid nitrogen and stored at -80°C. Blood samples were drawn immediately before the surgical intervention in EDTA-containing BD Vacutainer^®^ tubes and leukocytes were collected by centrifuging at 2.500 × *g* for 15 min at 4°C and stored at -80°C until use. The blood sample for DNA extraction was not available for one patient and the liver metastasis and cancer-free liver tissues were not available for another patient. DNA was extracted from both leukocytes and from tissue samples by a standard phenol/chloroform DNA extraction, as previously described (Udali et al., 2015). DNA concentration and purity were assessed by NanoDrop 1000 spectrophotometer (Thermo Fisher Scientific, Wilmington, DE, United States) and DNA was stored at -20°C.

### Global DNA mCyt and hmCyt Quantification

The mCyt and hmCyt content of genomic DNA extracted from leukocytes and tissue samples was determined by a liquid chromatography/mass spectrometry (LC/MS/MS) method, as previously described (Friso et al., 2002; Tammen et al., 2014a,b). Genomic DNA was hydrolyzed to nucleosides using 2 units of nuclease P1, 0.002 units of venom phosphodiesterase I and 0.5 units of alkaline phosphatase and isotope-labeled internal standards for the cytosine (Cyt) and for the modified cytosines (mCyt and hmCyt) were added to the hydrolyzed DNA. All experiments were performed on an Applied Biosystems 3200 Q Trap mass spectrometer system equipped with a turbospray ionization source (Applied Biosystem, Foster City, CA, United States). The liquid chromatograph used was an Agilent 1100 Series. A Suplex pKb 100 analytical column protected by a 5-μm Suplex pKb 100 precolumn (Supelco, Bellefonte, PA, United States) was used.

The mobile phase for all reactions consisted of 7 mM ammonium acetate pH 6.7/HPLC grade-methanol 5% (v/v) and was prepared with HPLC-grade water (all reagents from Fisher Scientific, Pittsburgh, PA, United States). The stable isotope-labeled compound [^15^N_3_]2′-deoxycytidine was used as internal standards for 2′-deoxycytidine (Cyt) and the custom-made (methyl-d_3_, ring-6-d_1_)-5-methyl-2′-deoxycytidine (both from Cambridge Isotopes Laboratories, Inc., Andover, MA, United States) was used as internal standards for 5-methyl-2′-deoxycytidine (mCyt) and 5-hydroxymethyl-2′-deoxycytidine (hmCyt) residues. Data was collected in multiple reaction monitoring (MRM) mode, using the mass transitions after ion fragmentation as follows: 2′-deoxycytidine at *m/z* 228.1→112.1, 5′-methyl-2′-deoxycytidine at *m/z* 242.1→126.1, and 5′-hydroxymethyl- 2′-deoxycytidine at *m/z* 258.1→142.1.

The absolute mass of Cyt, mCyt, and hmCyt was calculated using the intensity and the known mass of the internal standards. Global DNA methylation and hydroxymethylation were measured in relation to total cytosine. DNA methylation was expressed as % mCyt = [(mCyt)/(mCyt + hmCyt + Cyt)] × 100, whereas DNA hydroxymethylation was expressed as % hmCyt = [(hmCyt)/(mCyt + hmCyt + Cyt)] × 100 (Friso et al., 2002; Tammen et al., 2014a,b).

### Statistical Analysis

Continuous variables are expressed as the mean values ± standard deviations (SD). When variables showed a non-Gaussian distribution (e.g., hmCyt), the values were log-transformed and expressed as geometric means with 95% confidence intervals (CIs). Student’s paired *t*-test was applied to for pair-wise comparison of hmCyt and mCyt content between two different tissues from the same patient. Possible correlations between hmCyt and mCyt in the different tissue types were evaluated by calculating Pearson’s correlation coefficient. The correlation analysis was also performed between leukocytes and colon/liver tissues for both mCyt and hmCyt levels. All analyses were performed using IBM SPSS 20.0 statistical software (IBM, Inc., Armonk, NY, United States) and a *p*-value < 0.05 was considered significant.

## Results

### Clinical and Biochemical Characteristics of the Patients

The main clinical and biochemical characteristics of the patients affected by colon cancer and synchronous liver metastasis are reported in **Table 1**. The group included nine males and seven females with a mean of 66.3 ± 11.4 years of age, among whom, five patients reported a smoking habit, i.e., four former and one current smoker. Despite the presence of liver metastasis, patients did not show impaired liver function, as demonstrated by the liver indexes reported in **Table 1**.

**Table 1:** Clinical and biochemical characteristics of cancer patients.

		Reference values	Cancer patients (*n* = 16)
**Clinical characteristics**			
	Age (years)		66.3 ± 11.4
	Gender (male/female)		9/7
	BMI (kg/m^2^)	18–25	26.7 ± 3.7
	Smoking		
	Never		10 (66.7%)
	Former		4 (26.7%)
	Current		1 (6.7%)
	Diabetes mellitus type 2		4 (26.7%)
**Biochemical characteristics**			
	CRP (mg/L)^∗^	<5.00	12.1 (4.4–33.0)
	Hb (g/dL)	13.5–16	12.4 ± 1.3
	MCV (fL)	86.0–98.0	88.9 ± 4.8
	Platelets (10^9^/L)	150–400	297 ± 86
	White Blood Cells (10^9^/L)^∗^	4.30–10.00	6.78 (5.85–7.86)
	Glucose (mmol/L)^∗^	3.5–5.5	5.72 (4.76–6.88)
	AST (U/L)^∗^	5.0–50.0	51.1 (31.9–81.9)
	ALT (U/L)^∗^	6.0–50.0	40.2 (22.2–72.7)
	ALP (U/L)^∗^	50.0–130.0	132.2 (88.9–196.7)
	CHE (U/L)	5000–17000	6067 ± 1641
	GGT (U/L)^∗^	4.0–60.0	92.1 (45.2–187.7)
	Total bilirubin (mg/dL)^∗^	0.11–1.05	0.57 (0.43–0.76)
	Direct bilirubin (mg/dL)^∗^	<0.35	0.20 (0.11–0.35)
	INR^∗^	0.82–1.14	1.07 (1.00–1.14)
	Folate (nmol/L)^∗^	6.7–33.9	26.2 (13.8–49.6)
	Vitamin B6 (nmol/L)^∗^	25–128	16.4 (11.3–23.6)
	Vitamin B12 (pmol/L)^∗^	142–724	286 (207–395)
	Homocysteine (μmol/L)	<15	12.9 ± 4.5
	Ferritin (mg/L)^∗^	30–400	135 (66–279)
	Albumin (g/L)	35.0–50.0	36.4 ± 6.7

*^∗^Log-transformed variables are shown as geometric mean with 95% confidence interval.*

*CRP, C reactive protein; Hb, hemoglobin; MCV, mean cell volume; AST, aspartate aminotransferase; ALT, alanine aminotransferase; GGT, gamma-glutamyltranspeptidase; ALP, alkaline phosphatase; CHE, cholinesterase; INR, international normalized ratio.*

### Global DNA mCyt and hmCyt Content in Cancer-Free Tissue

**FIGURE 1:**
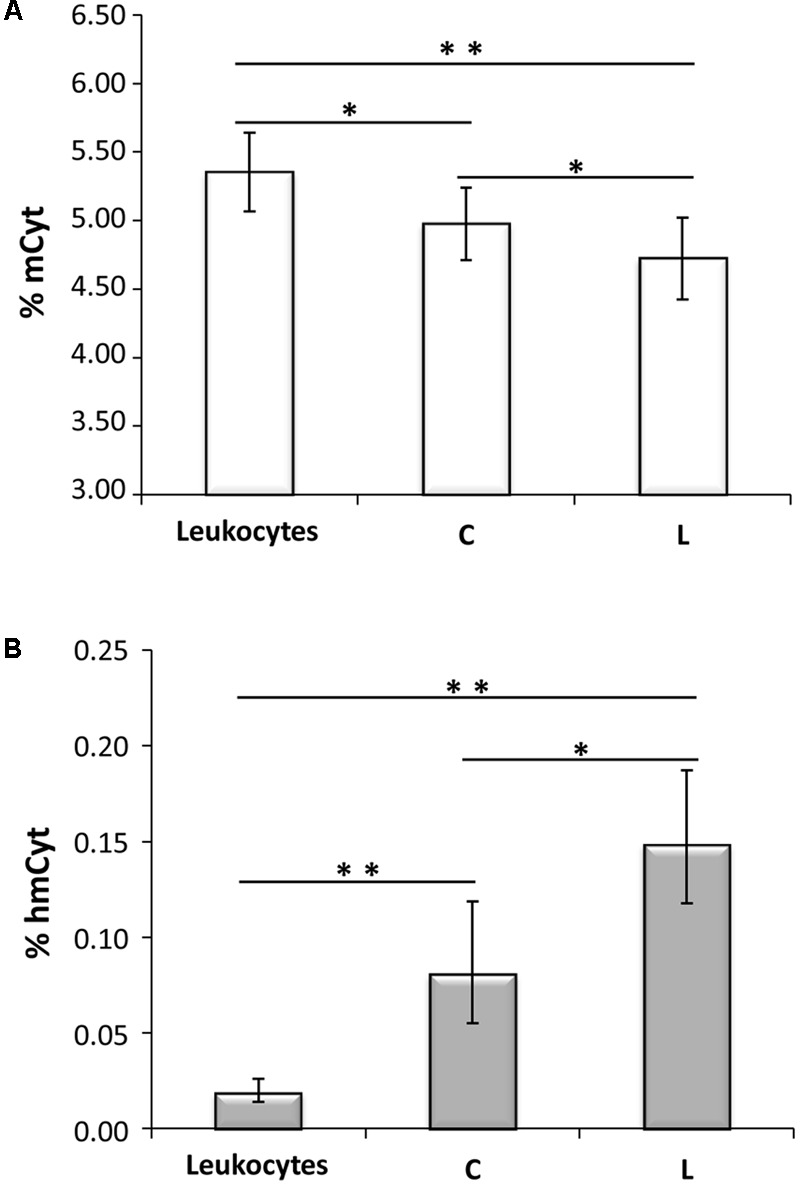
**(A)** Methylcytosine (mCyt) content differs in cancer-free tissues; **(B)** hydroxymethylcytosine (hmCyt) content is highly variable among the different cancer-free tissues. C, cancer-free colon tissue; L, cancer-free liver tissue; ^∗^*p* ≤ 0.05, ^∗∗^*p* ≤ 0.0001.

Methylcytosine and hmCyt contents were determined in the cancer-free specimens collected from the cancer patients, i.e., leukocytes, cancer-free colon (C), and cancer-free liver (L), to demonstrate possible differences due to tissue type. Methylcytosine levels were 5.35 ± 0.29% in leukocytes, 4.98 ± 0.26% in C and 4.72 ± 0.30% in L (**Figure 1A**). The differences among the three tissue types were statistically significant, i.e., leukocytes vs. L *p* < 0.0001, leukocytes vs. C *p* = 0.002, C vs. L *p* = 0.016 (**Figure 1A**). **Figure 1B** reported hmCyt levels that were highly variable among the different cancer-free tissue types. In particular, hmCyt content was strongly reduced in leukocytes, with a value of 0.019% (C.I. 0.014–0.026%), while the hmCyt value was 0.081% (C.I. 0.055–0.119%) in cancer-free colon and 0.149% (C.I. 0.118–0.187%) in cancer-free liver. The Student’s paired *t*-test showed significant differences with a *p*-value < 0.0001 in the comparison of leukocytes both with C and with L and a *p*-value of 0.010 in the comparison of C with L.

### Methylcytosine Content in Primary Colon Cancer and Synchronous Hepatic Metastasis

**FIGURE 2:**
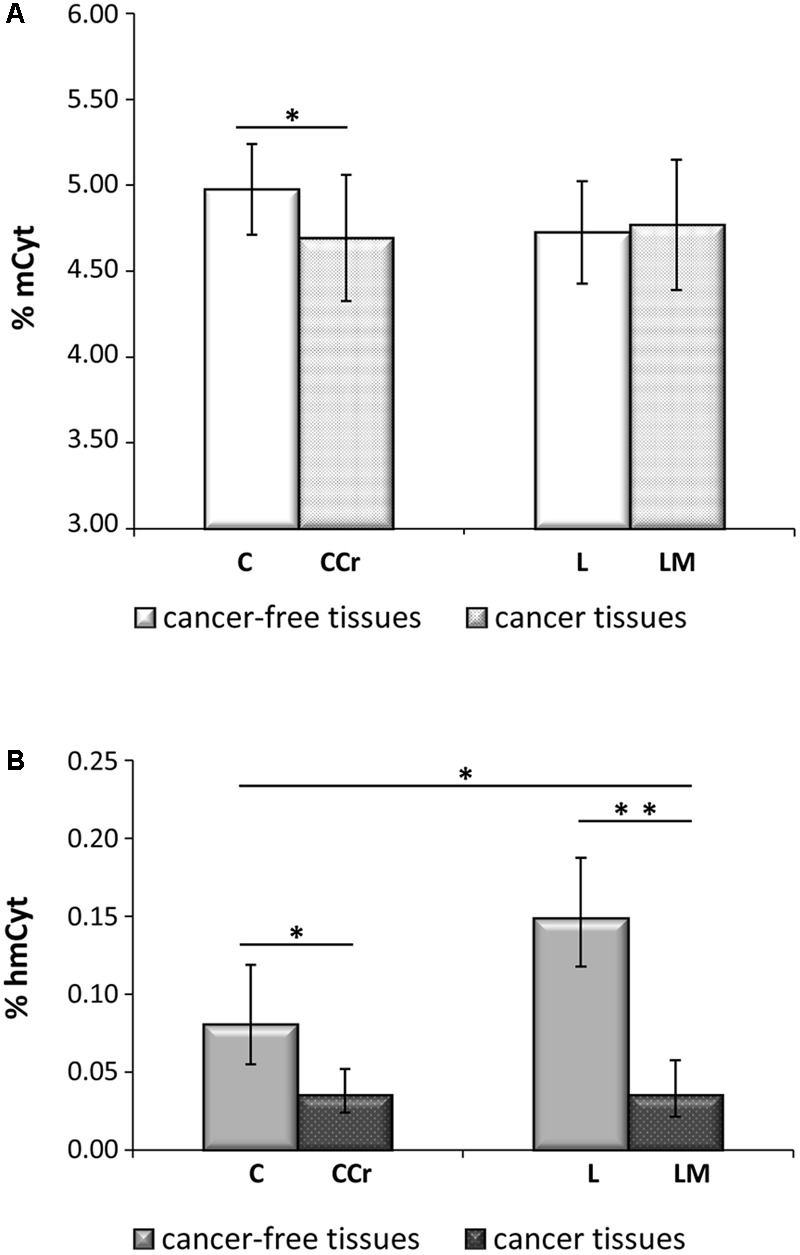
**(A)** Primary colon cancer (CCr) shows mCyt levels comparable with that of synchronous liver metastasis (LM) that are lower than cancer-free colon tissues (C). **(B)** hmCyt levels are comparable between primary colon cancer and synchronous liver metastasis and are significantly depleted compared to cancer-free colon (C) and cancer-free liver (L) tissues. ^∗^*p* ≤ 0.05; ^∗∗^*p* ≤ 0.0001.

In **Figure 2A**, colon cancer and metastatic tissues were compared to the homologous colon and liver cancer-free tissues. Liver metastatic tissues showed a mCyt content comparable to that of primary colon cancer (LM = 4.77 ± 0.38% vs. CCr = 4.69 ± 0.37%, *p* = 0.535). Methylcytosine levels were significantly lower in colon cancer compared to cancer-free colon, i.e., CCr = 4.69 ± 0.37% vs. C = 4.98 ± 0.26%, *p* = 0.004; mCyt content in liver metastasis tissue was lower than in cancer-free colon (4.77 ± 0.38% vs. 4.98 ± 0.26%, respectively), but the difference did not reach statistical significance (*p* = 0.148). As for mCyt content, no statistically significant differences were detected in the comparison of primary colon cancer tissue and cancer-free liver tissue (CCr = 4.69 ± 0.37 vs. L = 4.72 ± 0.30%, *p* = 0.879) and in the comparison of liver metastasis and cancer-free liver (LM 4.77 ± 0.38% vs. L = 4.72 ± 0.30%, *p* = 0.706) (**Figure 2A**).

### Hydroxymethylcytosine Content in Primary Colon Cancer and Synchronous Hepatic Metastasis

Primary colon cancer and synchronous liver metastasis showed analogous hmCyt content that was considerably reduced compared to homologous cancer-free tissues (**Figure 2B**). In particular, hmCyt content was 0.035% (C.I. 0.024–0.052%) in colon cancer and 0.081% (C.I. 0.055–0.119%) in cancer-free colon tissue (*p =* 0.002). When regard to synchronous liver metastasis, hmCyt content was 0.035% (C.I. 0.021–0.058%) and was significantly reduced compared to both cancer-free colon (C = 0.081% C.I. 0.055–0.119%, *p* = 0.008) and to cancer-free liver (L = 0.149%, C.I. 0.118–0.187%, *p* = 0.0001) (**Figure 2B**).

### Correlation Analyses for mCyt and hmCyt Levels

**FIGURE 3:**
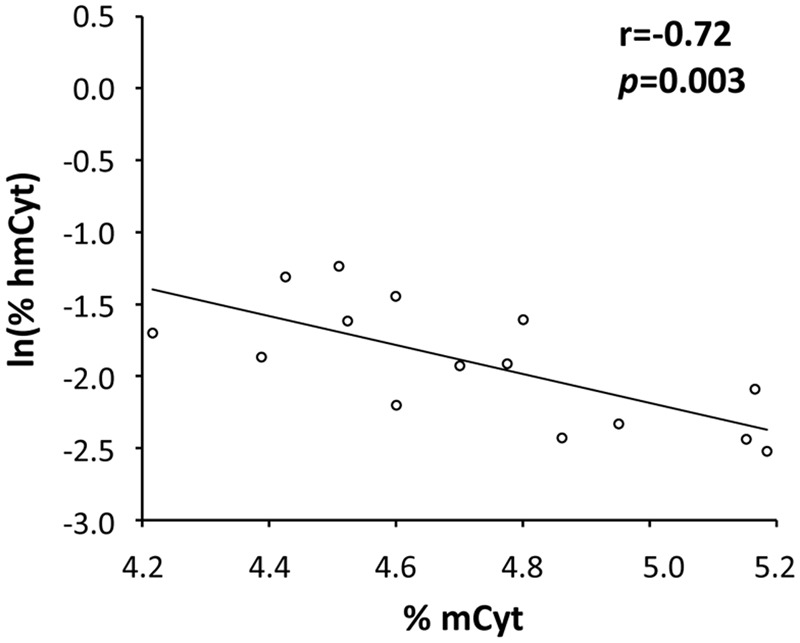
A statistically significant negative correlation between hmCyt and mCyt levels was observed in cancer-free liver tissue with a Pearson’s correlation coefficient (r) of –0.72 and a *p*-value of 0.003.

The possible correlations between mCyt and hmCyt were evaluated in the different tissue types to investigate the relationship between these two epigenetic markers. A significant negative correlation between mCyt and hmCyt was observed in the cancer-free liver tissue (Pearson’s correlation coefficient = -0.72, *p* = 0.003) (**Figure 3**). No correlations between mCyt and hmCyt emerged in leukocytes, cancer-free colon, primary colon cancer, and liver metastasis tissues.

**FIGURE 4:**
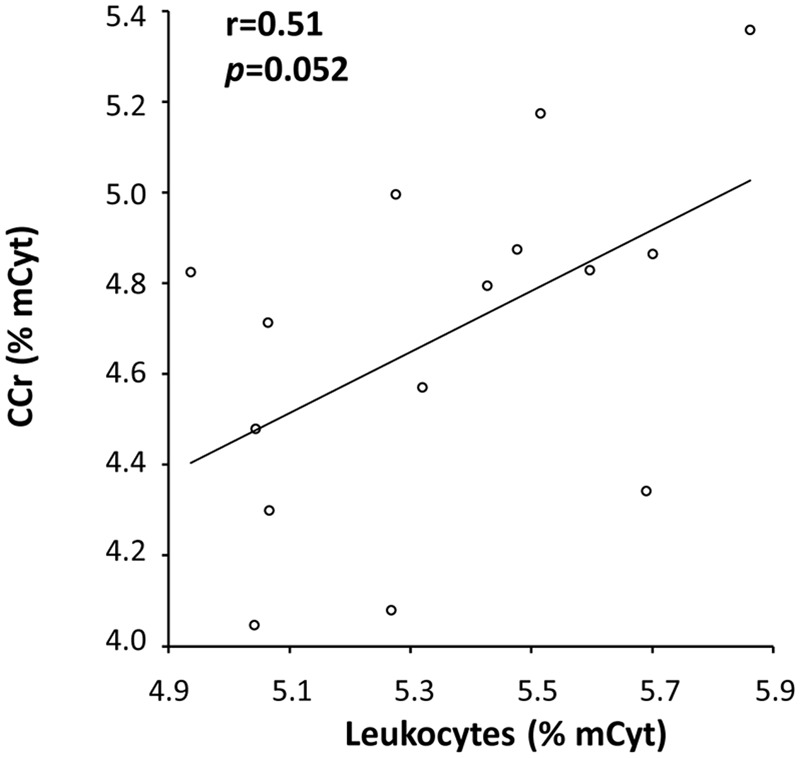
A positive correlation with a Pearson’s correlation coefficient (r) of 0.51 between mCyt levels in colon cancer tissue and mCyt levels in leukocytes was observed with a borderline *p*-value of 0.052.

A correlation analysis was also performed for mCyt and hmCyt levels between leukocytes and colon/liver tissues. Methylcytosine levels in leukocytes correlated with mCyt levels in colon cancer with borderline statistical significance (Pearson’s correlation coefficient = 0.51, *p* = 0.052) (**Figure 4**). No correlation was evident for the comparison between leukocytes mCyt levels and other tissue types, regardless of whether they were cancerous or cancer-free tissues. With regard to hmCyt levels, no statistically significant correlation was observed when comparing leukocytes with the other tissues evaluated in the present study.

## Discussion

Methylcytosine and, more recently, hydroxymethylcytosine levels, are gaining traction as two epigenetic marks of particular interest in cancer research both for their functional role in cancer development and their possible application as diagnostic and prognostic tools. Despite the increasing interest in this field, many aspects remain to be clarified since there are little data on the pattern of DNA methylation and DNA hydroxymethylation at a tissue level in primary cancer and metastatic tissues compared to non-affected tissues of the same organ affected by neoplasia together with leukocyte DNA data from the same cancer affected patients. In this study, patients were carefully characterized for clinical and biochemical conditions, as well as for the availability of DNA extracted from all of the different tissues involved in the disease, namely the site of cancer, i.e., colon, liver metastatic tissue, in addition to tissues from both colon and liver that were not affected by the cancer according to histological analyses. Furthermore, leukocyte DNA was also available for each patient. The simultaneous analysis of global DNA methylation and hydroxymethylation in primary and secondary colon cancer has been not yet been reported. It could help define the epigenetic landscape of metastatic colon cancer and highlight possible novel epigenetic markers from peripheral blood. The opportunity to compare homologous tissues allowed us to avoid possible bias due to the individual epigenetic variability among patients. Moreover, the LC–MS/MS method used for our analysis is the gold standard method for global DNA methylation quantification (Lisanti et al., 2013) and permits a sensitive and accurate quantification of both global DNA methylation and hydroxymethylation (Tammen et al., 2014a,b).

In this study, we observed that the primary colon cancer and liver metastasis tissues had a similar epigenetic status with significantly lower mCyt and hmCyt levels compared to homologous cancer-free colon and liver tissues.

Interestingly, in either colon and liver cancer-free tissues, we observed a significantly different content of both modified cytosines between specimens, suggesting that mCyt and hmCyt content depends on tissue type, as previously observed (Li and Liu, 2011; Udali et al., 2015). In particular, leukocytes showed the highest mCyt and the lowest hmCyt levels. In contrast, the liver presented the lowest mCyt and the highest hmCyt levels. When considering mCyt content for the comparison of cancerous versus cancer-free tissues, colon cancer tissue was significantly hypomethylated compared to cancer-free colon. The reduced mCyt content in colon cancer tissue substantiates the hypothesis that global hypomethylation could be a characteristic of this type of cancer (Feinberg et al., 1988; Hernandez-Blazquez et al., 2000; Zhang et al., 2011, 2013). However, some researchers did not observe a DNA hypomethylation status in colon cancer tissue (Tang et al., 2015). Similarly, we did not detect a hypomethylated status in other cancer tissues, including cholangiocarcinoma, a primary liver cancer type (Udali et al., 2015). Moreover, the majority of studies on global DNA methylation analyzed LINE-1 methylation status in colorectal cancer (Bariol et al., 2003; Sunami et al., 2011). LINE-1 regions are highly repeated regions in the human genome that are silenced through DNA methylation. LINE-1 hypomethylation has been associated with genomic instability in cancer (Kazazian, 2004). LINE-1 methylation status is used as a surrogate of the genome-wide methylation status, but as it is a sequence-specific methylation status, it is not easily comparable to the global methylation defined by the mCyt content quantified by mass spectrometry-based methods.

Very few studies have analyzed global DNA methylation in the context of colon cancer and synchronous metastasis by analyzing LINE-1 methylation status. Data from such studies are somewhat conflicting. Some authors detected identical methylation levels in colon cancer and in synchronous metastasis (Matsunoki et al., 2012; Murata et al., 2013), similarly to our present results while Hur and colleagues observed significantly lower LINE-1 methylation in liver metastasis compared to colon cancer tissues (Hur et al., 2014). Our findings and those by others (Matsunoki et al., 2012; Murata et al., 2013) suggest that hypomethylation may occur at an early stage in colon cancer development and be maintained even at the metastatic stage of cancer cells spread.

We also performed correlation analyses of mCyt levels in different tissues. We observed a positive correlation between mCyt levels in leukocytes and mCyt levels in colon cancer, with borderline statistical significance. These data are somewhat in agreement with those reported by Barciszewska et al. (2014) for brain tumors, although they evaluated methylation status from whole blood DNA. The borderline statistical significance observed in the present study may be due to the limited number of samples. The analysis of a larger sample size could be helpful to confirm these preliminary data.

With regard to global hydroxymethylation, the finding of a profound loss of hmCyt in cancer tissues is consistent with previous reports in a variety of human cancers, such as colorectal, prostate, breast, liver and lung cancers (Haffner et al., 2011; Jin et al., 2011; Li and Liu, 2011; Kudo et al., 2012; Zhang et al., 2013). Previous observations of high hmCyt levels in differentiated cells and, in contrast, low levels in tissue progenitor cells, suggest a special role for hmCyt in biological differentiation (Haffner et al., 2011; Tan and Shi, 2012). Consequently, hmCyt loss, which appears to be a peculiar characteristic of neoplastic tissues, could reflect the dedifferentiated state of cancer cells and their increased replicative capacity (Lee et al., 2015). The exact role of hmCyt in carcinogenesis is still the object of current investigations, but there are great expectations regarding the possible applications of global loss of hmCyt as a cancer biomarker for early diagnosis and prognosis (Lian et al., 2012; Yang et al., 2013).

Finally, we analyzed the possible correlation between mCyt and hmCyt levels in different tissues and observed an inverse correlation between the two modified cytosines that was only evident in the cancer-free liver tissue. There is no unequivocal explanation for such a finding, although it could be because in cancer-free tissue there is a clearer difference between the methylation and hydroxymethylation status. However, further analysis on a larger sample set could help to clarify the correlation between these two epigenetic markers.

## Conclusion

Primary colon cancer and synchronous liver metastasis tissues showed a similar epigenetic status opening up to the hypothesis of the occurrence of DNA hypomethylation and hypohydroxymethylation at the early stage of cancer development and its maintenance up to the phase of liver metastasis progression of colon cancer disease. Moreover, the finding of a correlation between mCyt levels in leukocytes and mCyt levels in colonic cancer tissue may suggest that leukocyte DNA methylation parallels that of the tissue methylation status of genomic DNA. These observations indicate a possible role for leukocyte DNA methylation as a cancer biomarker and suggest the need of deepening the understanding on this yet open issue in the field of cancer epigenetics.

## Author Contributions

SU, DD, ST, and PP collected samples and performed the experiments. AR, SM, FM, GB, and TC enrolled the patients, analyzed the data and revised the manuscript. OO and AG coordinated the research, interpreted the data and revised the manuscript. S-WC and SF designed and coordinated the research. SU and SF analyzed the data and wrote the paper. All the authors participated in discussion and editing of the manuscript and approved the final version of the manuscript.

## Conflict of Interest Statement

The authors declare that the research was conducted in the absence of any commercial or financial relationships that could be construed as a potential conflict of interest.
